# Visual Tooth Color Determination with Different Reference Scales as an Exercise in Dental Students’ Education

**DOI:** 10.3390/dj11120275

**Published:** 2023-11-29

**Authors:** Thomas U. Klinke, Wolfgang B. Hannak, Klaus Böning, Holger A. Jakstat, Elisabeth Prause

**Affiliations:** 1 Department of Prosthodontics, Gerodontology and Dental Materials, Center of Oral Health, University Medicine Greifswald, 17489 Greifswald, Germany; 2 Charité, Center for Dental and Craniofacial Sciences, Department of Prosthodontics, Geriatric Dentistry and Craniomandibular Disorders, Campus Benjamin Franklin, 12203 Berlin, Germany; wolfgang.hannak@charite.de (W.B.H.); elisabeth.prause@charite.de (E.P.); 3 Department of Prosthodontics, Carl Gustav Carus Faculty of Medicine, Technische Universität Dresden, 01069 Dresden, Germany; klaus.boening@uniklinikum-dresden.de; 4 Department of Prosthodontics and Material sciences, University Leipzig, 20251 Leipzig, Germany; holger.jakstat@medizin.uni-leipzig.de

**Keywords:** student’s dental education, color competence, tooth guide, color determination, shade taking

## Abstract

Visual color determination (VCD) requires color competence and individual training. The aim of this study was to evaluate the deviations in students’ VCD with two different reference scales. The research hypothesis was that none of the color references would provide a better result. Participants evaluated nine templates randomly using two reference scales (VITA-classical (VC) and 3D-Master-Toothguide (3DM_TG)). The color distance to the chosen color (ΔE_ab_) was calculated in the CIELAB 2000. The sum’s changes in the parameters (LCh°) represented the target variable. Results were evaluated with non-parametric, rank-scaled methods, utilizing the median with a 25%-75% quartile. The significance level (α = 0.05) is determined using the Student’s *t*-test. The mean ± 95%CI (SD) was −1.27 ± −1.09 (3.18); the median ΔE_00_ was −1.49 (−1.97; 0.96) for dC_3DM_TG_. The determination with VC showed noticeable differences (dC_VC_), with a mean ΔE_00_ of 0.00 ± 0.00 (2.20) and a median ΔE_00_ of 0.00 (1.17; 1.71). The standard error was 0.19 for the dC_VC_ and 0.27 for the dC_3DM_TG_. dC_3DM_TG_ vs. dC_VC_ showed significant differences at *p* < 0.001. The dental student’s VCD resulted in color deviations, regardless of the reference template used. The color deviations in hue and chroma were comparable, regardless of the reference scale. VCD’s early implementation in dental education is useful to avoid shade misjudgments and potentially expensive remakes of dentures.

## 1. Introduction

Patient satisfaction and acceptance of a dental restorative, prosthodontic fixed, or removable denture are closely related to personal approval and positive expectations regarding the treatment’s outcome. This pertains not only to achieving proper functionality but also to aesthetic appearance. In this context, the reconstruction’s color and, particularly, color determination play a pivotal role. Shade determination is necessary for the reproduction and characterization of artificial restorations and represents a daily challenge for practitioners in dental practice [1,2].

Visual and/or instrumental methods are currently used in dental offices to determine the shade of teeth. They serve as a basis for shade communication with the dental technician and are later the basis for shade control of dental prostheses. The tooth shade is visually matched via direct and continuous comparison of the natural tooth (or “template” in scientific studies) with the corresponding shade reference [3,4]. The aim of color determination is to determine the reference pattern that represents the smallest possible color deviation, i.e., the least perceived color discrepancy [1,5,6,7,8,9,10]. However, it must be stated here that in visible color determination, the reference templates do not always precisely match the tooth to be determined. 

The composition of the color references varies and corresponds to the most frequently occurring empirically determined tooth colors in nature. The manufacturer-specific reference scales differ from each other in the empirical selection of colors with color coordinates (L* a* b* values) in the color space, as well as in the material and distribution of the reference samples on the color scale [11]. A “true, visual color match” with a reference sample is subjectively burdened and can lead to more or less visible, significant deviations due to individual color perception [5,11]. Dental prostheses’ color corrections are time-consuming, involve additional effort and costs, require further patient appointments, and may ultimately result in resource-intensive and cost-incurring reconstruction of the dental prostheses [12,13]. In a study, Lehmann et al. investigated the accuracy and reproducibility of visual and digital color determination. They concluded that an accuracy of 72.5% and a reproducibility of 48.0% can be assumed for visual color determination [14]. The subjective perception of the observer varies significantly and depends on parameters such as age, gender [15,16,17], education [18,19,20,21,22,23,24,25,26] and profession (prior knowledge) [22,25,26,27,28,29,30,31]. Studies show the extent of subjective color perception [26,32,33,34].

**Figure 1:**
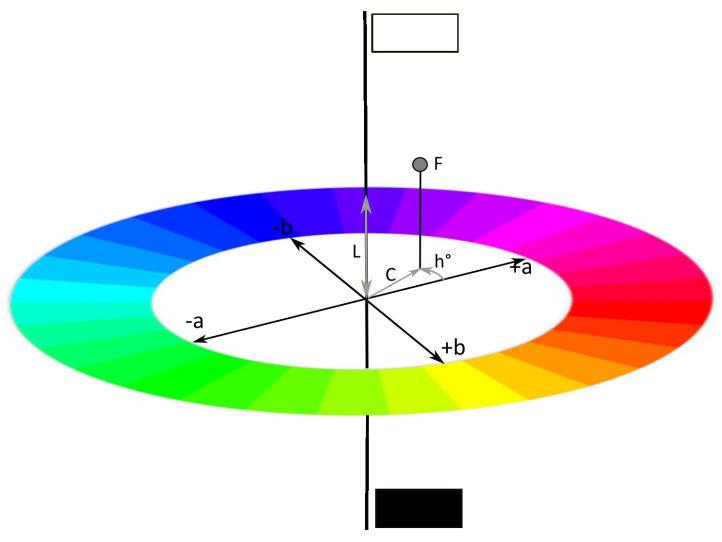
The CIE-LCh system. The localization of the point “F” can be specified as the color angle (hue value, H in h°) and vector length C* (chroma) of the color saturation in the cylinder coordinate (L*, C*, and h°).

The Cartesian coordinates L*, C*, and h° are used for the systematic description of the color parameters brightness (lightness, L*), color intensity (chroma, C*), and hue (h°) [3,35,36,37]. The brightness (L*) describes two poles in the spherical color space: “white” represents the imaginary north pole, while “black” occupies the imaginary south pole, and all gray-tone values are arranged in the vertical imaginary connection line. All other saturated, chromatic hues (h°) are represented at the equator of the sphere, with the axes in the equatorial plane, the a*- and b*-axes, representing the color valences of red–green and blue–yellow, respectively (Figure 1).

The conversion of Cartesian coordinates (L*, a*, and b*) into the mentioned cylindrical coordinates (L*, C*, and h°) is carried out using Formulas (1)–(3) [38].

(1) $$L_{a,b}=L$$

(2) $$C_{a,b}=\sqrt{a^{2}+b^{2}}$$

(3) $$h^{{}^{\circ}}{}_{a,b}=\mathrm{atan}\left(\frac{b}{a}\right)$$

To perceive color differences (ΔE), colors in the color space should ideally be arranged equidistantly. The color difference between two colors is described as the distance between the involved locations (L1, a1, and b1 as well as L2, a2, and b2) and is calculated using the Euclidean distance Formulas (4) and (5), although distributional asymmetries of colors in this system are known [13,14,35,36,37,38,39].

(4) $$\Delta E_{\mathrm{ab}}=\sqrt{{\left(L1-L2\right)}^{2}+{\left(a1-a2\right)}^{2}+{\left(b1-b2\right)}^{2}}$$

(5) $$\Delta E_{\mathrm{ab}}=\sqrt{\Delta L^{2}+\Delta a^{2}+\Delta b^{2}}\;$$

For each chromatic color and its mixed color, the corresponding angle h° is given: 0° to 90° for colors between red and yellow, between 90° and 180° for colors between yellow and green. The colors between green and blue are described with the angle h° between 180° and 270°, and the angle between 270° and 360° represents the colors from blue to red. [10,39,40] Regardless of the parameters used (Lab* or L*C* h°), differences can be measured and scientifically evaluated [6,7,8,9,41,42].

An improvement in the cognitive abilities and visual color differentiation skills of the dentist or user is possible with appropriate training sequences [23,31,43,44,45]. The authors used templates that were matched in training with the appropriate reference scale [22,23,26,45,46,47,48,49,50]. McMaugh examined the differentiation ability of different cohorts: undergraduates (first and fourth clinical years), dentists and esthetic dentistry specialists, and dental technicians [22]. He found no difference between the results of the cohort of students from different semesters. Lack of student instruction and practice is discussed as a cause of the result [22]. A significant difference was found between the cohort of first-year students and specialists. He based his result on the experience of the student cohort and the enthusiasm with which a specialist implements his focus in his work. The difference between the dentist cohort and the specialists was explained by the fact that the dentists were overwhelmed by the large number of different reference shades and limited themselves to only three to four “standard shades” in their daily practice [28]. Without doubt, the good result of the dental technician cohort (dental ceramists) was due to the intensive examination of the tooth shade and the continuous checking of the (working) result. Capa et al. found in their study with dentists, dental staff, and students that experience and expertise improve and increase tooth shade determination results [28]. Haddad et al., on the other hand, could not find a significant difference in the results in the study group between the subject groups (dentists (*n* = 295) and students (*n* = 319)) and the level of experience [16]. The study group around Nakhaei et al. concurred with this result under the condition that a special reference scale (VITA 3D-Master, V3D M) was used [31]. The differences when using another reference shade guide (VITA Classical, VC) led to significant differences between the cohorts [31].

Haddad et al. demonstrated the influence of age and gender on tooth color matching in their international study with 614 participants (305 females and 309 males) from 15 universities. They found that female participants achieved significantly better results in tooth color differentiation than male participants [16]. Based on their findings, they concluded that females play an important role in shade selection and shade matching. Furthermore, in this research, they found that the level of experience does not play a crucial role in color matching. However, Haddad et al. disagree with the research of Miranda, who found that males and participants had a significantly better result in color differentiation than females [17]. Furthermore, Haddad et al. contradicted the studies of Ristic et al. and Olms et al., who found that participants achieved significantly better results in tooth color matching after participating in a curriculum of tooth color matching [49,51]. Here, the demand was postulated that color differentiation should be implemented in the training in order to avoid large color deviations, which result in a new supply or a costly improvement [49].

Capa et al. also found a positive correlation with the result, while gender and the use of visual aids (glasses or contact lenses) had no influence on the determination of tooth shade [28]. Without doubt, the good result of the dental technician cohort (ceramists) was due to the intensive examination of the tooth shade and the continuous color checking in the working progress. The authors concluded and postulated that tooth color determination should be conducted by clinicians only who participated in continuing education programs and other training courses on color competence. This can increase experience and expertise and improve tooth color identification results [28].

In summary, Wee et al. referenced studies that explored the perceptibility of a color difference, denoted as ΔEab [37,52]. Delta E (∆E) defines the perceived color distance, indicating the difference between two colors; the index “ab” refers to the reference quantity, designating the color space (CIE Lab). This measurement can be used for quantifying works related to colors. Several studies have investigated the smallest color distance still distinguishable by the average viewer (“usual user”) [53]. As a result, both ΔE = 1 and ΔE = 2.5 were found. (Table 1) Various influencing factors significantly impact visual color matching, with adherence to the observation time being one of them. 

To obtain reliable results in evaluating color deviations, specific conditions must be precisely defined. These conditions include illuminance, adaptation time to illumination, and the color or brightness of the near and far surroundings in the viewing field. To ensure consistent and accurate conditions, visual sampling should be conducted in sample booths that adhere to these defined conditions [47].

Color differences (ΔE_ab_) ranging from ΔE_ab_ = 1 [36] to 2.54 [32] and ΔE_ab_ = 2.72 [55] to 3.8 [56] were found in vitro, and ΔE_ab_ = 3.7 to 6.8 [55] were found in vivo. Baltzer et al. showed that ΔE_ab_ values of 1 to 3 were required, and higher ΔE_ab_ values of 3 to 6 are considered suboptimal [3]. (Table 1) The “perceptibility threshold” and “acceptance threshold” are crucial factors in this context. The thresholds for color differentiation differ between groups of individuals (dentists, dental students, dental assistants, dental technicians, and laypeople): the 50:50% perceptibility thresholds (ΔE_ab_ = 1.2 or ΔE_00_ = 0.8) and the 50:50% acceptance thresholds (ΔE_ab_ = 2.7 or ΔE_00_ = 1.8) [57]. These threshold values can serve as control instruments and have been implemented in the ISO standard of 2016 [57,58].

The aim of this study was to investigate the suitability of two reference scales for determining a template’s color. The null hypothesis is that none of the reference scales used leads to a significantly better result in the VCD of untrained participants.

## 2. Materials and Methods

**Figure 2:**
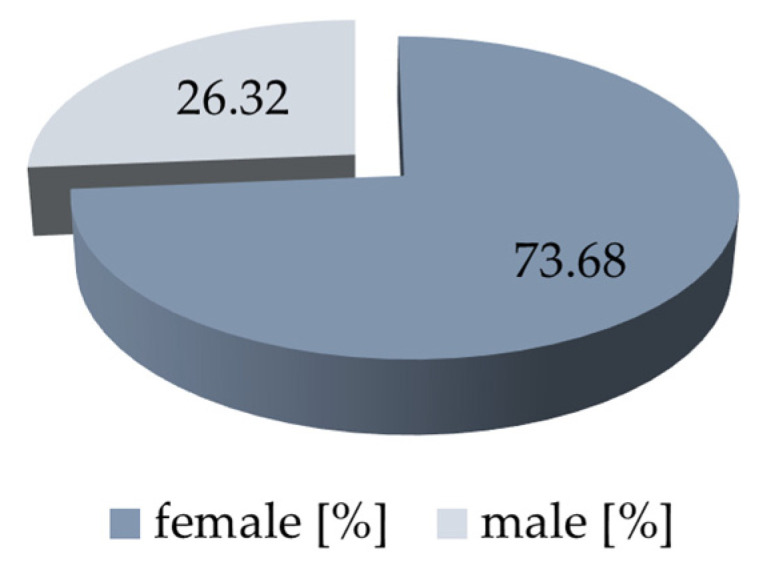
Gender distribution in the proband’s cohort [%].

Preclinical dental students (female *n* = 28 and male *n* = 10, Figure 2) from the University Medical Center Leipzig, the University of Dresden, the University of Berlin, and the Greifswald University participated in the study. The participants should be enrolled dental students; no further specific inclusion or exclusion criteria were formulated. The study participants voluntarily took part in the curriculum for tooth shade determination, which is offered annually at the participating universities as an additional course to the curriculum. Prior to study participation, the subjects underwent a color vision screening using the Ishihara test. For the screening of color blindness (Congenital Color Vision Deficiency (CCVD)), the Ishihara color charts were displayed on smartphone screens for the first time on Science Day, and the results were tested for sensitivity and specificity in comparison to the classic display on the PC monitor. These results are currently being summarized in a further publication [59].

**Figure 3:**
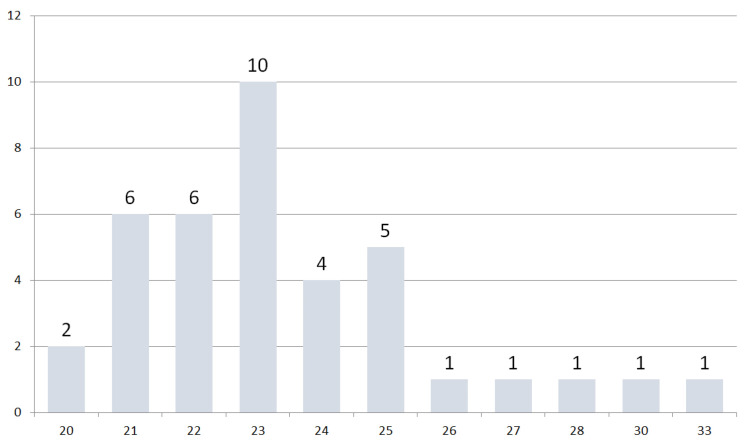
The distribution of participants’ ages has a mean of 23.5 ± 2.65 years.

The average age was 23.5 years ± 2.65 years, with a median age of 23.0 ± 13.0 years. (Figure 3).

The study was reviewed and approved by an Ethics Committee at the University of Greifswald (BB 175/22). The participants evaluated 9 randomly selected templates (incisors of another reference scale (Chromascop^®^ Shade guide, Ivoclar Vivadent, Schaan (Liechtenstein)) twice using two different reference scales (VITA classical (VC) and 3D-Master Toothguide (3DM TG), both from VITA Zahnfabrik, Bad Säckingen, Germany). The VITA 3D-Master Toothguide reference scale was used to improve the comparability of the results. The linear arrangement of the reference patterns was examined by Paravina et al. and leads to a comparable, intuitive template’s matching procedure [48]. The templates were measured in advance with a calibrated spectrometer (VITA EasyShade V, VITA Zahnfabrik). The tooth shade determination was carried out under clinical conditions, i.e., neutral ambient conditions were ensured for reproducible tooth shade determination. This included uniform illumination of the environment with daylight lamps (brightness: 1750 lumens; color temperature: 4500 K) [30,60]. 

Differentiation was performed in a seated position at a viewing distance of 25 to 35 cm, following the recommendations of Cordel et al. [12]. The evaluation included the color distance of the template to the chosen pattern in the L* ab color space and was converted into the CIELAB 2000 color space [61]. The distance (ΔE_ab_) was calculated using the Euclidean formula (4), the result was summarized, and the mean was calculated for both groups. The changes in the sum of the differences for each participant represented the target variable. The differences (ΔE) within the used parameters (L*C h°) were evaluated and subjected to evaluation. The results were evaluated using a statistical program (SPSS v21, SPSS Inc., Chicago, IL, USA) with non-parametric, rank-scaled methods using the median and the 25%- and 75%-quartile. The Student’s *t*-test was used to verify the significance level (α = 0.05).

## 3. Results

**Figure 4:**
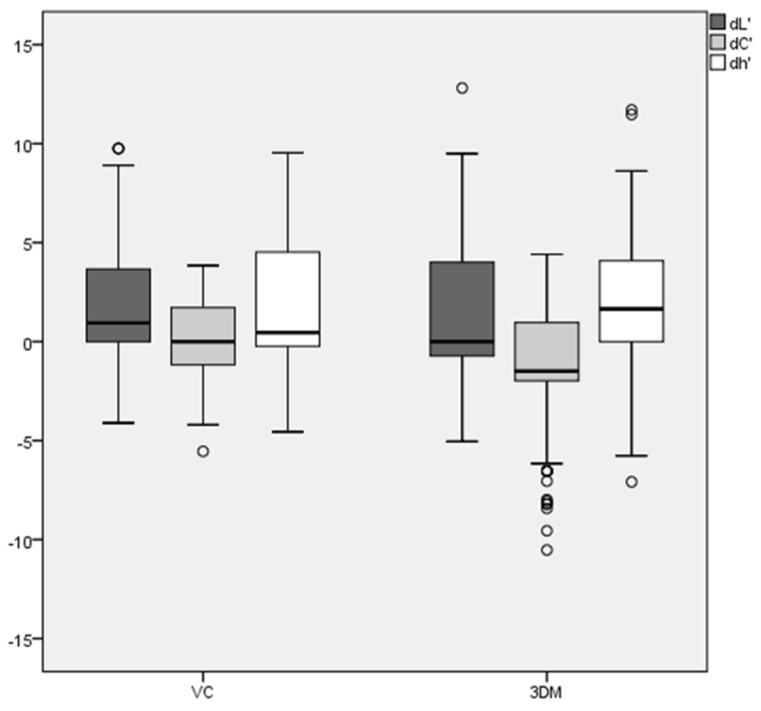
Deviation (ΔE_00_) for color determination in hue value (h°), lightness (L), and vector length C* (chroma) with VITA Classical (VC) and with VITA 3D–Master-Toothguide (3DM) reference scale of student’s color determination.

The overall results for the 3DM TG reference scale showed for the parameters dL_3DM TG_, with a mean ΔE_00_ ± 95% confidence interval (standard deviation, SD) of 0.9 ± −1.04 (3.1) and a median ΔE_00_ of 0.0 (25%-; 75%-quantile: −0.7; 4.0) for VC. The determination using the VC reference scale showed noticeable color differences in dL_VC_, with a mean ΔE_00_ of 1.4 ± 1.05 (3.1) and a median ΔE_00_ of 0.94 (25%-; 75%-quantile: 0; 3.7). The standard error (SE) was comparable between the dL_VC_ group (0.27) and the 3D-Master group (dL_3DM TG_ 0.26). (Figure 4) The differences (*t*-test) in the results of both reference scales were not statistically significant (*p* = 0.683).

For the parameters dh°_3DM TG_, there were noticeable color differences, with a mean of 1.68 ± −1.3 (3.83) and a median ΔE_00_ of 1.64 (25%-; 75%-quantile: 0; 4.84) for dh°_3DM TG_. The determination using the VC reference scale showed noticeable color differences dh°_VC_, with a mean ΔE_00_ of 1.59 ± 1.11 (3.26) and a median ΔE_00_ of 0.45 (25%-; 75%-quantile: −0.45; 4.84). The standard error (SE) was approximately comparable between the dh°_VC_ group (0.28) and the 3D-Master TG group (dh°_3DM TG_) (0.33) (Table 2).

The differences (*t*-test) in the results of both reference scales were not statistically significant; dL’ *p* = 0.196 and dh’ *p* = 0.839, but dC’ showed significant differences (*p* < 0.001). The mean was −1.27 ± −1.09 (3.18), and the median ΔE_00_ was −1.49 (25%-; 75%-quantile: −1.97; 0.96) for dC_3DM TG_. The determination using the VC reference scale showed noticeable color differences in dC_VC_, with a mean ΔE_00_ of 0.00 ± 0.00 (2.20) and a median ΔE_00_ of 0.00 (25%-; 75%-quantile: −1.17; 1.71). The standard error (SE) was 0.19 for the dC_VC_ group and 0.27 in the 3D-Master group (dC_3DM TG_).

## 4. Discussion

Within the limitations of this study, the research hypothesis that there were no differences in the color differentiation results of the shade guides used (VITA Toothguide 3D-Master vs. VITA Classical) that demonstrated superior performance in determining the shade of a template tooth was not rejected. Determining the tooth shade is a crucial step in daily dental practice, and existing restorations near the tooth to be restored can complicate shade differentiation due to its chameleon effect. Consequently, the shade of the existing tooth may not be accurately represented on the shade scale, and the most suitable and closest tooth shade is selected to minimize shade deviation [12]. Numerous articles and studies have shown that experts (dentists, dental technicians) can determine tooth shade more accurately than patients, with shade deviations of ΔE < 2 being visually perceptible and recognizable [27,55].

In this present study, the VITA 3D-Master TG reference color scale yielded better results, possibly due to its broader color space coverage resulting from a higher number of references (*n* = 29) and a more evenly distributed, equidistant arrangement in the color space [57]. On the other hand, the VITA Classical reference scale, with only 16 color templates and an empirically random distribution of colors, led to faster but more challenging decisions. For better method comparison, Paravina et al. advocated a linear arrangement of shade samples, which was intuitively used by the expert group (dental technicians and dentists) [33]. The color arrangement in the reference scale’s tab leads the user intuitively and quickly to a result in direct comparison. Paravina et al. see the reason for this in the physiology of color vision. Thus, the authors assume that in color vision, a separation of brightness, saturation, and hue is not possible for the human eye, but rather a color impression consisting of all three-color dimensions and their color interactions is perceived [62]. 

One limitation of this study that needs to be discussed is that the results were limited to a cohort of young dental students. These students were in the pre-clinical training phase and were only taught about tooth color determination in a lecture. Up to this point, they had no clinical experience with patients. Even though McMaugh found no differences between the cohort groups of preclinical students (first and fourth year students), he did find that the misjudgment of tooth color became significantly lower in the postgraduate phase [22]. He explained this improvement by citing the increasing clinical experience of practitioners and the enthusiasm and precision with which restorative dentistry specialists in particular approach shade selection [22]. Capa et al. also found that the cohort comparison of dentists, dental assistants, and dental technicians achieved better results in tooth shade selection compared to students [28]. For this reason, Paravina et al. argued that teaching tooth shade differentiation should be introduced at a later stage in the dental curriculum [44]. Another limitation of the study could be the number of participants. Although the number of study participants should always be higher, the cohort studied here consists of students who voluntarily took part in the annual “Science Day of the Universities”.

A further limitation of the present study is the proband’s number. The results of this research are based on a cohort of preclinical students who were instructed in the performance of clinical tooth shade differentiation in a lecture. As with all clinical studies, the results should not only be broadly based, but the cohort should also have different levels of prior knowledge. In terms of gender distribution, it should be noted that almost three-quarters of the participants were female (74%), and only a quarter of the participants were male (26%). In further studies in this area of tooth shade determination, attention should also be paid to the equal distribution of the two gender groups in order to be able to work out a gender difference.

Another aspect that needs to be discussed concerning the results presented here is the use of VITA 3D-Master TG. Although the participants were instructed in the use of the reference scale in advance, in contrast to the use of the VITA Classical reference scale, the VITA 3D-Master TG shade guides the user through a step-by-step approximation of the tooth shade selection. In this process, after determining the color brightness (five color gradations), the color intensity (five categories) is determined. Finally, as the last step, the shade (in the yellowish or reddish range with three categories) is determined. Through the determination of lightness (value) at the first level, up to 60% of incorrect assessments are eliminated. Both further steps, the determination of chroma and hue, reduce color-matching errors with a compliance of almost 70% [33,34]. 

A curriculum during training is recommended as an essential factor for success within tooth shade differentiation [23,43,44,45,46,51,63]. There is agreement on the fact that an improvement of cognitive abilities and skills in visual color differentiation is possible through adequate training [45,47,49,50,64]. At this point, Paravina et al. stated that courses in which information about color with prosthetic or conservative emphasis, color science, and the fundamentals of color vision; the influence of environmental parameters such as color-matching lamps (color-rendering index); and color references (VITA 3D-Master Bleached Guide) are considered useful [48]. He considered the appropriate time for the curriculum to be in the post-graduate phase rather than in pre-clinical training [48].

However, the evaluation method used in this present study did not establish the superiority of the VITA Toothguide 3D-Master over the VITA Classical (*p* > 0.05), leading to the acceptance of the null hypothesis. It should be discussed whether the better results with the VITA Toothguide 3D-Master stem from its closer proximity to the template’s color. Another contributing factor could be the equidistant color pattern distribution of the VITA Toothguide 3D-Master in the color space, enabling better representation of color nuances compared to the VITA Classical reference scale.

The target variable in this study focused on color deviation from the original: larger L* a b coordinate differences resulted in large deviations, while narrow L* a b coordinates resulted in smaller deviations in ΔE from the template. This demonstrates the sufficiency of the subjects’ color choices and the suitability of the color references used in each case.

Visual shade matching of adjacent teeth with a shade reference remains the standard method for shade selection, despite its susceptibility to inhomogeneity. The adequacy of matching the natural tooth color in direct visual matching depends on factors like viewing distance and the distribution of colors in the reference scales. It is reasonable to assume that the sought-after color may not be exactly represented in any available reference shade guide. No significant difference was found in color differentiation when using two color reference scales for subject decisions. Similar results obtained with the evaluation method used here need further investigation [65].

In dental practice, electronic spectrophotometers significantly increase the accuracy of color determination compared to visual methods. They offer stability in results, reducing the impact of local measurement conditions and handling variability. To minimize subjective variance and ensure consistency in tooth shade determination, electronic instrumental aids have gained popularity in dental practices. The digital spectrophotometer, such as the VITA Easyshade® (VITA Zahnfabrik, Bad Säckingen, Germany), serves as a reference standard for tooth shade determination in clinical studies [8,42,66,67,68]. Various scientific studies report reliability rates of 87.4% to 99.0% and accuracy rates of 67% to 93% for digital dental colorimeters [69,70]. Despite some deviations in calculated color coordinates from the spectrophotometric reference system, the instrumental color measurement demonstrated excellent repeatability [6]. For daily practice, however, an additional visual inspection and validation of the instrumental color measurements is still recommended.

## 5. Conclusions

From this, the following can be concluded and postulated:

- In view of patients’ increasing desire for highly esthetic dentures, tooth shade determination should be part of dental training;
- In order to reduce misjudgments in tooth shade determination, training and instruction in tooth shade differentiation should be implemented in dental training or in an additional curriculum;
- The kind of reference scale used does not play a role in tooth shade determination training;
- Rather, the differentiation result improves through application in the dental practice or through additional training.

## Figures and Tables

**Table 1:** Interpretation of color distances ΔE as Euclidean distances of L*a*b* values or from polar coordinates L*C*h* [32,54].

ΔE	Valuation
0.0–0.5	exact match/no difference in color
0.5–1.0	very good match/small difference, visible for a trained eye
1.0–2.0	non-recognizable color difference, good match/acceptable
2.0–4.0	recognizable color difference, poor match
4.0–5.0	noticeable color difference, hardly acceptable
>5.0	mismatch/totally unacceptable, difference will be evaluated as a different color

**Table 2:** Distribution of the Cartesian coordinates L*, a*, and b* as a function of the reference scales used, which are used for the systematic description of the color parameters lightness (L*), color intensity (chroma, C*), and hue (hue, h°).

	VITA 3DM TG			VC		
	dL	dC	dh°	dL	dC	dh°
**Mean ± SD**	0.97 ± 3.06	−1.27 ± 3.18	1.68 ± 3.83	1.45 ± 3.09	−0.00 ± 2.20	1.59 ± 3.26
**Confidence interval (95% CI)**	0.45–1.49	−1.81–−0.72	1.03–2.33	0.93–1.98	−0.37–0.37	1.03–2.14
**Median**	0.00	−1.49	1.64	0.94	0.00	0.45
**Standard error (SE)**	0.26	0.27	0.33	0.27	0.19	0.28

## Data Availability

The data presented in this study are available on request from the corresponding author. The data are not publicly available due to restrictions, e.g., privacy.
